# The association between body mass index and lower extremity deep venous abnormalities identified by computed tomography venography

**DOI:** 10.3389/fpubh.2026.1842325

**Published:** 2026-08-05

**Authors:** Yan-Qin Lan, Zhi-Feng Xi, Kai-Sen Lan, Xiao-Ying Huang, Ning Feng, Ying-Min Chen

**Affiliations:** 1 Medical Imaging Department, Hebei General Hospital, Shijiazhuang, Hebei, China; 2 Operation Department, Hebei General Hospital, Shijiazhuang, Hebei, China; 3 Office of NCD and Ageing Health Management, Chinese Center for Disease Control and Prevention, Beijing, China

**Keywords:** body mass index, computed tomography venography, lower extremity deep vein, stenosis, thrombosis

## Abstract

**Objective:**

To investigate the association between body mass index (BMI) and lower-extremity deep venous (LEDV) abnormalities, including thrombosis and stenosis, identified by computed tomography venography (CTV), with analysis on sex-specific patterns and the influence of hypertension, diabetes, and hyperlipidemia.

**Methods:**

This retrospective cross-sectional study analyzed 349 patients (217 males, 132 females) undergoing CTV. Patients were stratified by sex, age, BMI, and comorbidities (hypertension, diabetes, hyperlipidemia). The per-patient counts of thrombosis and stenosis were compared using *t*-tests. Multivariable logistic regression (reporting adjusted odds ratios) and negative binomial regression (reporting adjusted incidence rate ratios) were used to assess the independent associations of BMI with LEDV prevalence and lesion burden, adjusting for age, sex, comorbidities, and major venous thromboembolism risk factors. The threshold for statistical significance was set at *P* < 0.05.

**Results:**

Overweight or obesity was highly prevalent among patients with LEDV abnormalities (86.4% of males, 79.0% of females). Males had more LEDV stenoses than females (1.19 vs. 0.77 per person, *P* < 0.05). In females, coexisting overweight/obesity with hypertension, diabetes, and/or hyperlipidemia was associated with higher thrombosis prevalence (*P* < 0.05), which was not observed in males; for stenosis, this comorbidity effect was present in both sexes and more pronounced in males. In multivariable logistic regression, no factor, including BMI was independently associated with LEDV prevalence (all *P* > 0.05). By negative binomial regression, male sex was independently associated with greater stenosis count (aIRR 1.65, 95% CI 1.19–2.28, *P* = 0.002) and overall lesion burden (aIRR 1.32, *P* = 0.048); hypertension was borderline (aIRR 1.39, *P* = 0.05); BMI was not.

**Conclusion:**

Higher BMI was associated with a greater prevalence of CTV-detected LEDV abnormalities, particularly stenosis and in the presence of comorbidities. After multivariable adjustment, however, BMI was not an independent predictor; male sex was the principal independent correlate of stenosis burden, with hypertension borderline. These cross-sectional associations are hypothesis-generating.

## 1 Background

Venous disease, encompassing both venous thromboembolism (VTE) and lower-extremity deep venous (LEDV) disease, is strongly associated with obesity. Epidemiologic evidence indicates that obese individuals are significantly more likely to develop symptomatic lower-limb venous disorders, with about 25% already carrying a formal diagnosis and many others exhibiting unrecognized symptoms (1).

Overweight and obesity are also well-established contributors to a broad spectrum of non-communicable chronic diseases (NCDs), including hypertension, cardiovascular disease, type 2 diabetes, hyperlipidemia and so on (2–5). LEDV thrombosis and stenosis are progressive vascular disorders caused by coagulation abnormalities, endothelial damage, and reduced venous flow, pathologies that can result in severe complications such as pulmonary embolism and post-thrombotic syndrome, substantially impairing quality of life (6–10). Despite the biological plausibility of this connection, robust, data-driven evidence on the direct relationship between body weight and specific LEDV abnormalities such as thrombosis and stenosis remains limited.

To quantitatively investigate this relationship, body mass index (BMI) serves as a critical and measurable determinant of risk. A UK population study reported that individuals with a BMI exceeding 30 kg/m^2^ were more than twice as likely to develop venous reflux, a precursor to chronic venous disease, highlighting BMI as a central risk factor for LEDV (1). The clinical impact of an elevated BMI extends beyond mere incidence, affecting outcomes across conditions; for instance, the prognosis of chronic venous disorders deteriorates progressively with increasing BMI, with patients exceeding 35 kg/m^2^ showing reduced therapeutic response to interventions (11). While the hypercoagulable state in obesity is well-documented (12–16), and some studies have examined postoperative LEDV thrombosis (17, 18), a crucial gap exists: few studies have directly assessed the relationship between BMI and the prevalence of LEDV thrombosis and stenosis in a general patient population using advanced imaging.

This relationship is unlikely to be uniform and is potentially complicated by a key biological variable: sex. Epidemiologic studies consistently demonstrate that women have a higher cumulative incidence of varicose veins and elevated rates of deep vein thrombosis (DVT) during their reproductive years, whereas DVT prevalence in men rises progressively with age (19, 20). Critically, obesity appears to amplify this inherent disparity, with the risk of DVT increasing more sharply in women than in men, with reported hazard ratios of 2.75 and 2.02, respectively (21). These observations suggest that sex not only shapes biological susceptibility to venous pathology but also modulates the interaction between obesity and thrombotic risk. Consequently, these findings underscore the necessity of sex-stratified analyses to move beyond a one-size-fits-all understanding.

To conduct such a precise, sex-stratified investigation, an advanced imaging modality capable of comprehensive assessment is required. computed tomography venography (CTV) has become an invaluable tool for this purpose. It offers crucial advantages specifically in visualizing centrally located thrombi in the superior vena cava and right atrium (22). Its diagnostic utility is particularly pronounced for identifying iliac vein obstruction (IVO) and May-Thurner syndrome (MTS) (23, 24). CTV identified 79% of LEDV abnormalities that much higher than the percentage detected by color Doppler ultrasound, with a higher prevalence of severe stenosis observed in males (22). This ability to provide a detailed anatomical map makes CTV a convenient, noninvasive, and highly informative method for guiding surgical decision-making (22, 25) and managing complex cases (24).

Despite extensive evidence linking obesity to deep vein thrombosis (DVT) and related venous disorders, knowledge gaps persist, revealing a need for a more nuanced investigative approach. First, while sex differences in DVT incidence are acknowledged, previous studies have not fully elucidated the sex-specific difference details such as degree, quantity, or spectrum, that is the manifestation of differences in different situations, as well as mechanisms underlying these differences. Furthermore, the interactions between body mass index (BMI) and comorbid CVD in modulating thrombotic risk remain poorly defined (12, 21). Second, from a diagnostic standpoint, the complementary limitations of CTV and ultrasonography, where CTV's superiority in detecting central thrombi contrasts with ultrasound's peripheral strength, highlight the necessity of leveraging advanced imaging for a comprehensive characterization of venous pathology in obese populations (24).

Addressing these specific gaps requires a targeted, sex-stratified, CTV-based analysis to clarify the complex interplay between obesity, CVD, and sex-specific physiology in LEDV development. Therefore, this study leveraged a substantial dataset of CTV-diagnosed cases from a large population of hospitalized patients to investigate the association between BMI and the prevalence of LEDV thrombosis and stenosis. The data was analyzed by systematically stratifying by sex, age, and key comorbidities, including hypertension, diabetes, and hyperlipidemia. Through this approach, we aim to enrich the evidence base for targeted, sex-sensitive prevention and management strategies.

## 2 Methods

### 2.1 Study design

This was a cross-sectional quantitative study. All medical records of patients with body weight and height and LEDV status through CTV examination at Hebei General Hospital of China from April 1, 2017 to November 30, 2024 were selected and reviewed. The inclusion criteria were: (1) hospitalization at the hospital during this period; (2) documented body height and weight at admission; and (3) a completed lower extremity CTV examination. The exclusion criteria were maternity (pregnancy or the peripartum period) and May-Thurner syndrome.

The following information of the patients were copied to a specially designed statistical table for the study by the research team: (1) Sex, age, body weight (kg) and height (m) at hospitalization; (2) Whether or not suffered from comorbidities (hypertension, diabetes and/ or hyperlipidemia); (3) Diagnosis at discharge; (4) Whether or not suffered from LEDV thrombosis, the sites (which LEDV and part) and number of the thrombosis identified by CTV; (5) Whether or not suffered from LEDV stenosis, the sites and number of the stenosis identified by CTV.

The LEDVs included common iliac vein (CIV), internal iliac vein (IIV), external iliac vein (EIV), common femoral vein (CFV), popliteal vein (POV), anterior tibial vein (ATV), posterior tibial vein (PTV), and peroneal vein (PEV). The total number of thrombosis and stenosis in LEDV for each patient were calculated. A single venous segment can be coded as both thrombosed and stenosed, and the two lesions were counted and analyzed separately.

Both male and female patients were divided into 3 age groups as 20~ year-old, 40~ year old and 60~ year-old ones. They also were classified as different body weight groups as low (L, BMI <18.5kg/m^2^), moderate (M, 18.5 kg/m^2^ ≤ BMI <24.0kg/m^2^), overweight (Ov, 24.0kg/m^2^ ≤ BMI <28.0kg/m^2^), obese (Ob, BMI ≥ 28.0kg/m^2^) ones according to BMI standard used in China.

Among all subjects (*n* = 349), overweight and obese individuals with hypertension and/or diabetes and/or hyperlipidemia simultaneously were classified as Group A. Among the remaining subjects, people without hypertension, diabetes or hyperlipidemia were classified as Group B.

The average number of thrombosis and/or stenosis per capita were applied to describe the prevalence of the thrombosis and/or stenosis in LEDV. *T*-test was applied to compare means.

Multivariable analyses were performed (*n* = 349). Binary prevalence outcomes (presence vs. absence of LEDV thrombosis, stenosis, and thrombosis and/or stenosis) were analyzed by logistic regression, with Firth penalization where sparse-data separation occurred, reporting adjusted odds ratios (aORs). The number of thrombosis/stenosis/combined per patient (lesion burden), which was over-dispersed, was analyzed by negative binomial regression, reporting adjusted incidence rate ratios (aIRRs). All models were simultaneously adjusted for age, sex, BMI, and hypertension, diabetes, and hyperlipidemia (entered as separate covariates), with documented major venous thromboembolism risk factors additionally included.

The statistical significance was set at *P* < 0.05.

CTV machine and measurement parameters in this study may refer to the authors' former articles (22, 24). Informed Consent was obtained from each patient before CTV testing.

### 2.2 Quality control

The research team developed a unified variable statistical table for this study. All variables were defined uniformly. Two researchers copied the data of each variable from the same medical record and into the statistical table and verified the data to guarantee their corrections. The data was entered double times. The two researchers also doubly checked the calculation of the numbers of the thrombosis and stenosis in LEDV for each included patient.

In actual clinical practice, each CTV report is composed by a qualified imaging physician who operates the CTV examination together with a facilitator, and is reviewed by a senior supervising physician (chief physician) before issuance. CTV examination physicians receive regular training on standardized operations. These measures ensure the quality of test results and consistency of diagnosis.

### 2.3 Confidentiality

The collected data in the study was only for the use of researchers' analysis that was not allowed to be disclosed to others. Meanwhile, the data was analyzed from a population but not individual perspective so that there was no individual data presented. Neither was the variable linking with personal information such as patient name, age, testing results or diagnosis etc.

### 2.4 Ethics and consent to participation

This study was a retrospective cross-sectional survey study based on existing medical records of discharged patients. Written Informed Consent was obtained from each patient before his/her CTV testing. So, this study conformed to the guidelines laid down in the Declaration of Helsinki. After reviewing, this paper conforms to the principals of medical ethics. It was accepted to publish by Hebei General Hospital Ethics Committee (No. 2025-LW-01116, April 15th, 2025). The data was accessed for research purposes during January 5–15, 2025, and the authors had not accessed to information that could identify individual participants during or after data collection.

## 3 Results

### 3.1 Basic information

A total of 349 patient records were included, comprising 217 males (62.2%) and 132 females (37.8%). They had a mean age of 57.97 ± 11.56 years (median 59, interquartile range <IQR> 52–66) and a mean BMI of 27.46 ± 4.00 kg/m^2^. Men had a mean age of 57.28 ± 11.97 years (median 58, IQR 51–66) and BMI of 27.75 ± 3.92 kg/m^2^, while women had a mean age of 59.11 ± 10.78 years (median 60, IQR 53–66) and BMI of 26.97 ± 4.08 kg/m^2^.

Meanwhile, among them, 5 patients (1.4%) had low body weight, 54 patients (15.5%) had moderate body weight, 146 patients (41.8%) were overweight, and 144 patients (41.3%) were obese. So, a total of 290 patients (83.1%) were overweight and obese. There were 30 (8.6%) patients in the age group of 20~ year-old, 148 (42.4%) patients in the age group of 40~ year-old, and 171 (49.0%) patients in the age group of 60~ year-old. As shown in Table 1.

**Table 1:** Sex, age, and body weight classification (number and percentage) of the included patients.

Age	Value type	Male					Female					Total				
		L	M.	Ov.	Ob	Sub-total	L	M.	Ov.	Ob	Sub-total	L	M.	Ov.	Ob	Sub-total
20 ys~	N	1	1	5	15	22	0	2	3	3	8	1	3	8	18	30
	%	4.5	4.5	22.7	68.2	100	0.0	25.0	37.5	37.5	100	3.3	10.0	26.7	60.0	100
40 ys~	N	2	8	40	45	95	1	15	19	18	53	3	23	59	63	148
	%	2.1	8.4	42.1	47.4	100	1.9	28.3	35.8	34.0	100	2.0	15.5	39.9	42.6	100
60 ys~	N	1	18	40	41	100	0	10	39	22	71	1	28	79	63	171
	%	1.0	18.0	40.0	41.0	100	0.0	14.1	54.9	31.0	100	0.6	16.4	46.2	36.8	100
Total	n	4	27	85	101	217	1	27	61	43	132	5	54	146	144	349
	%	1.8	12.4	39.2	46.5	100	0.8	20.5	46.2	32.6	100	1.4	15.5	41.8	41.3	100

There were 128 patients in Group A, accounting for 36.7% of all included patients. Among them, there were 77 males, accounting for 35.3% of male patients, and 51 females, accounting for 38.6% of female patients. There were 202 patients (128 males and 74 females) in group B, accounting for 57.9% of all included patients (Table 2).

**Table 2:** The number and percentage (cases, %) of Groups A and B^*^.

Age	Male		Female		Total	
	Group A	Group B	Group A	Group B	Group A	Group B
20 ys	3, 0.9	19, 5.4	0, 0	7, 2.0	3, 0.9	26, 7.5
40 ys	31, 8.9	59, 16.9	14, 4.0	37, 10.6	45, 12.9	96, 27.5
60 ys	43, 12.3	50, 14.3	37, 10.6	30, 8.6	80, 22.9	80, 22.9
Total	77, 22.1	128, 36.7	51, 14.6	74, 21.2	128, 36.7	202, 57.9

^*^%, with *n* = 349 as the denominator.

### 3.2 Prevalence and univariate analysis

There were 48 (22.1%) male patients suffered from LEDV thrombosis, of which 81.4% were overweight and obese ones, accounting for 18.0% of the male patients. There were 115 male patients (53.0%) suffered from LEDV stenosis, of which 87.0% were overweight or obese ones, accounting for 46.1% male patients. For females, 32 patients (24.2%) had LEDV thrombosis, with 78.5% being overweight or obese, accounting for 19.0% female patients; 67 cases (50.8%) suffered from LEDV stenosis, of which 78.9% were overweight and obese ones, and accounting for 40.1% females. Totally, 140 male patients (64.5%) suffered from LEDV thrombosis and/or stenosis, of which 86.4% were overweight and obese ones, accounting for 55.7% male patients. 81 female patients (61.4%) had LEDV thrombosis and/or stenosis, of which 79.0% were overweight and obese ones, accounting for 48.5% female patients (Table 3).

**Table 3:** Number (cases) and percentage (%) of patients with thrombosis and/or stenosis in LEDVs.

Abnormality	Value type	Male					Female					Total				
		L.	M.	Ov.	Ob.	Sub-total	L.	M	Ov.	Ob.	Sub-total	L.	M.	Ov.	Ob.	Sub-total
Thrombosis	n	1	8	21	18	48	0	7	15	10	32	1	15	36	28	80
	%	2.1	16.7	43.8	37.5	100.0	0.0	21.9	46.9	31.3	100.0	1.3	18.8	45.0	35.0	100.0
	%	0.5	3.7	9.7	8.3	22.1	0.0	5.3	11.4	7.6	24.2	0.3	4.1	9.8	7.6	21.7
Stenosis	n	1	14	47	53	115	1	13	32	21	67	2	27	79	74	182
	%	0.9	12.2	40.9	46.1	100.0	1.5	19.4	47.8	31.3	100.0	1.1	14.8	43.4	40.7	100.0
	%	0.5	6.5	21.7	24.4	53.0	0.8	9.8	24.2	15.9	50.8	0.5	7.3	21.4	20.1	49.3
Thrombosis and/or	n	2	17	58	63	140	1	16	37	27	81	3	33	95	90	221
	%	1.4	12.1	41.4	45.0	100.0	1.2	19.8	45.7	33.3	100.0	1.4	14.9	43.0	40.7	100.0
	%	0.9	7.8	26.7	29.0	64.5	0.8	12.1	28.0	20.5	61.4	0.9	9.5	27.2	25.8	63.3

The average number of LEDV thrombosis was 0.51 per person, with 0.49 per person in males and 0.55 per person in females. There was no statistically significant difference between male and female patients (*P* > 0.05). The average number of thrombosis per person in obese and overweight male patients were 0.54 and 0.37, respectively, which were lower than those in overweight (0.57 per person) and obese (0.53 per person) female ones without statistically significant difference (*P* > 0.05). The average number of thrombosis per person in LEDVs in male overweight and obese patients were lower than that in moderate weight patients, and higher in overweight patients than in obese ones, but neither of the difference was statistically significant (*P* > 0.05). There was no statistically significant difference in the average number of LEDV thrombosis among different age groups (*P* > 0.05). As shown in Table 4.

**Table 4:** Average number of the thrombosis and stenosis per capita in LEDV for different sex and age group patients.

Age	Male					Female					Total				
	L.	M.	Ov.	Ob.	Sub-total	L.	M.	Ov.	Ob.	Sub-total	L.	M.	Ov.	Ob.	Sub-total
Thrombosis															
**20 ys~**	0.00	0.00	0.80	0.47	0.50	NA	0.00	1.00	0.00	0.38	0.00	0.00	0.88	0.39	0.47
**40 ys~**	1.00	1.00	0.75	0.40	0.61	0.00	0.47	0.95	0.39	0.60	0.67	0.65	0.81	0.40	0.61
**60 ys~**	0.00	0.78	0.30	0.29	0.38	NA	0.70	0.36	0.73	0.52	0.00	0.75	0.33	0.44	0.44
15.6-7.4,-1.3690pt**Total**	0.50	0.81	0.54	0.37	0.49	0.00	0.52	0.57	0.53	0.55	0.40	0.67	0.55	0.42	0.51
Stenosis															
**20 ys**	1.00	5.00	0.60	1.33	1.32	NA	0.00	0.33	1.33	0.63	1.00	1.67	0.50	1.33	1.13
**40 ys~**	0.00	1.75	1.10	0.96	1.06	1.00	0.87	0.89	0.89	0.89	0.33	1.17	1.03	0.94	1.00
**60 ys~**	0.00	0.44	1.28^*^	1.68^*^	1.28	NA	0.50	0.85	0.50	0.69	0.00	0.46	1.06	1.27	1.04
15.6-7.4,-1.3690pt**Total**	0.25	1.00	1.15	1.31	1.19	1.00	0.67	0.84	0.72	0.77^#^	0.40	0.83	1.02	1.13	1.03
Thrombosis and/or stenosis															
**20 ys~**	1.00	5.00	1.40	1.80	1.82	NA	0.00	1.33	1.33	1.00	1.00	1.67	1.38	1.72	1.60
**40 ys~**	1.00	2.75	1.85	1.36	1.67	1.00	1.33	1.84	1.28	1.49	1.00	1.83	1.85	1.33	1.61
**60 ys~**	0.00	1.22	1.58	1.98	1.66	NA	1.20	1.21	1.23	1.21	0.00	1.21	1.39	1.71	1.47
**Total**	0.75	1.81	1.69	1.67	1.67	1.00	1.19	1.41	1.26^#^	1.31	0.80	1.50	1.58	1.55	1.54

^*^When compared with moderate-weight male patients of the same age group, *P* < 0.05; ^#^When compared with male patients of the same body weight type, *P* < 0.05.

On average, the number of stenosis in LEDV per person was 1.03 for all patients, and 1.19 and 0.77 for male and female patients respectively. Male patients had more stenosis in LEDV than female ones statistically significantly (*t* = 4.050, *P* < 0.05). The average number of stenosis per person was higher than that of thrombosis per person in LEDVs, and the differences in total and male patients were statistically significantly (*t*_total_ = 4.556, *t*_male_ *t* = 4.357, *P* < 0.05). The average number of stenosis per person in obese and overweight male patients were 1.15 and 1.31, respectively, which were higher than those in overweight (0.84/person) and obese (0.72/person) female ones. The differences between male and female obese patients was not statistically significant (*t*_obese_ = 1.866, 0.05 <*P* < 0.100), but the difference between the total male and female patients was significant (*t*_total_ = 2.074, *P* < 0.05). The average number of thrombosis per male overweight and obese patients in the age group of 60 years old was lower than that of moderate body weight group, and the differences were statistically significant (*t*_overweight_ = 3.069, *t*_obese_ = 1.987, *P* < 0.05). There was no statistically significant difference in the average number of stenosis per person in LEDVs between different age groups (*P* > 0.05). As shown in Table 4.

The average occurrence of LEDV thrombosis and/or stenosis was 1.54 per person, with 1.67 per person in male patients and 1.31 per person in female patients. Male patients had a higher occurrence than female ones without statistical significance (*t* = 1.718, 0.05 <*P* < 0.100). There was a statistically significant difference in the number of LEDV thrombosis and/or stenosis between obese male and female patients (*t* = 2.032, *P* < 0.05). There was no statistically significant difference in the average number of individuals with LEDV thrombosis and/or stenosis among different age groups (*P* > 0.05). As shown in Table 4.

**Figure 1:**
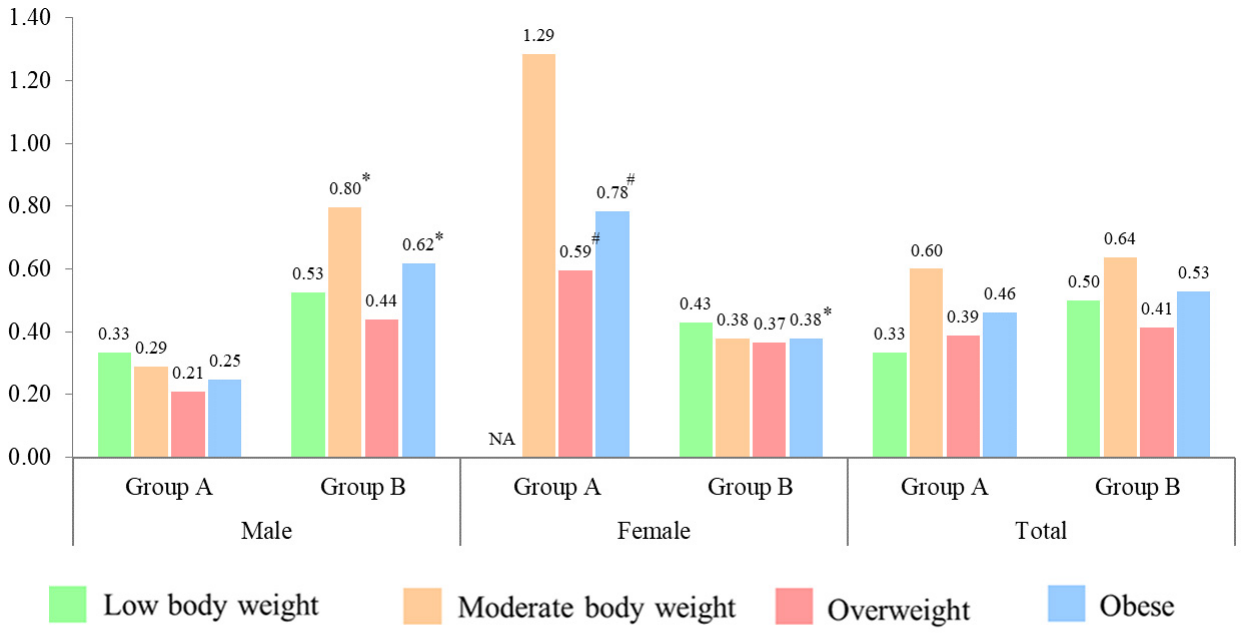
The comparison of average numbers of LEDV thrombosis between Groups A and B. *When compared with moderate-weight male patients of the same age group, *P* < 0.05; ^#^When compared with male Group A patients of the same age group, *P* < 0.05.

The average number of LEDV thrombosis in female Group A patients was higher than that in Group B ones, without statistically significant difference (*P* > 0.05). It was also higher than that in male Group A ones of the same age and the total age groups. The differences for 60~ year-old and the total age groups were statistically significant (*t*_60_ = 2.106, *t*_total_ = 2.072, *P* < 0.05). However, the average number of LEDV thrombosis in Group A males was lower than that in Group A ones, and the differences for 40~ year-old and the total age groups were statistically significant (*t*_40_ = 2.229, *t*_total_ = 2.835, *P* < 0.05). As shown in Figure 1.

**Figure 2:**
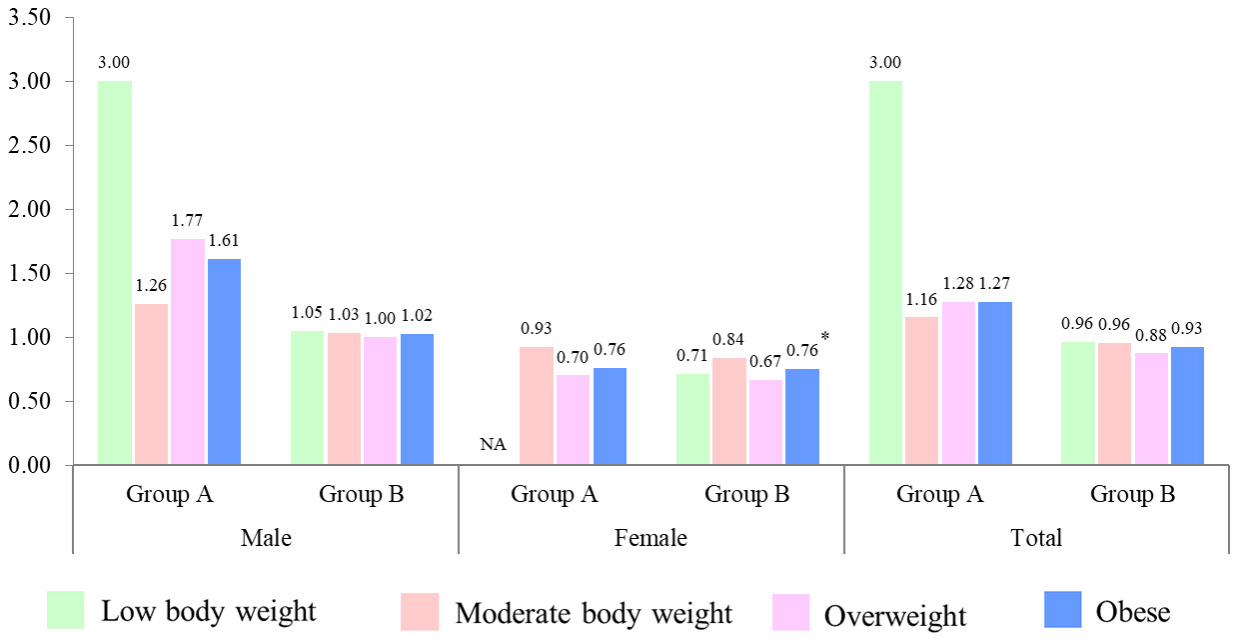
The comparison of average numbers of LEDV stenosis between Groups A and B. *When compared with male Group A patients, *P* < 0.05.

The average number of LEDV stenosis in male Group A patients was higher than that of Group B ones, but the difference was not statistically significant (*P* > 0.05). Female patients had similar comparative results. The average number of LEDV of male Group A patients was higher than that of female Group A ones, and the differences for 60~ year-old and the total age groups were statistically significant (*t*_60_ = 1.775, 0.05 <*P* < 0.100; *t*_total_ = 2.218, *P* < 0.05). As shown in Figure 2.

### 3.3 Multivariable regression analysis

In multivariable logistic regression, no factor achieved statistical significance (*P* > 0.05); the smallest *P*-values were for hypertension with stenosis (*P* = 0.128) and the documented major VTE risk factor with combined estimate (*P* = 0.074) but a wide confidence interval (Table 5).

**Table 5:** Multivariable logistic regression analysis of LEDV abnormality prevalence^*^.

Variable	Thrombosis (*n* = 80)		Stenosis (*n* = 182)		Thrombosis and/or stenosis (*n* = 221)	
	Adjusted ORs (95% CI)	*P*-value	Adjusted ORs (95% CI)	*P*-value	Adjusted ORs (95% CI)	*P*-value
Age (per + 1 year)^∧^	1.00 (0.98–1.02)	0.994	0.98 (0.96–1.00)	0.097	0.98 (0.96–1.00)	0.063
Male (vs. female)	0.91 (0.54–1.54)	0.734	1.14 (0.73–1.78)	0.577	1.18 (0.74–1.87)	0.487
BMI (per + 1 kg/m^2^)	0.97 (0.91–1.04)	0.417	0.99 (0.94–1.05)	0.773	0.99 (0.94–1.05)	0.787
Hypertension	0.87 (0.49–1.55)	0.645	1.46 (0.90–2.38)	0.128	1.14 (0.69–1.88)	0.62
Diabetes	1.68 (0.82–3.41)	0.155	0.85 (0.45–1.63)	0.632	1.48 (0.75–2.92)	0.262
Hyperlipidemia	1.32 (0.39–4.49)	0.657	0.55 (0.17–1.78)	0.318	0.54 (0.17–1.76)	0.305
Documented major VTE risk factor	1.57 (0.39–6.29)	0.522	3.58 (0.79–16.33)	0.099	14.83 (0.77–285.54)	0.074

^*^Firth penalization was used; the prevalence was presence/absence. ^**∧**^Covariate in the regression.

In negative binomial regression, male with stenosis (IRR 1.65, 95% CI 1.19–2.28) and combined counts (IRR 1.32, 95% CI 1.00–1.73) were statistically significant (*P* < 0.05), and hypertension with stenosis count (IRR 1.39, 95% CI 1.00–1.93) was marginally (*P* = 0.05) (Table 6).

**Table 6:** Negative binomial regression analysis of the abnormality counts.

Variable	Thrombosis count		Stenosis count		Combined count (thrombosis + stenosis)	
	Adjusted IRR (95% CI)	*P*-value	Adjusted IRR (95% CI)	*P*-value	Adjusted IRR (95% CI)	*P*-value
Age (per +1 year)^∧^	0.99 (0.97–1.02)	0.494	1.00 (0.98–1.01)	0.482	0.99 (0.98–1.01)	0.262
Male (vs female)	0.90 (0.52–1.56)	0.697	1.65 (1.19–2.28)^*^	0.002	1.32 (1.00–1.73)^*^	0.048
BMI (per +1 kg/m^2^)	0.98 (0.92–1.06)	0.655	1.00 (0.96–1.04)	0.947	0.99 (0.96–1.03)	0.673
Hypertension	0.73 (0.39–1.35)	0.309	1.39 (1.00–1.93)	0.050	1.14 (0.85–1.52)	0.390
Diabetes	1.92 (0.91–4.03)	0.085	0.82 (0.52–1.30)	0.398	1.20 (0.83–1.75)	0.336
Hyperlipidemia	1.10 (0.27–4.53)	0.897	1.55 (0.73–3.27)	0.254	1.30 (0.67–2.51)	0.442
Documented major VTE risk factor	1.13 (0.23–5.46)	0.881	1.60 (0.68–3.75)	0.279	1.36 (0.64–2.87)	0.425

^*^P < 0.05; ^∧^Covariate in the regression.

## 4 Discussion

The direct association between BMI and LEDV abnormalities, such as thrombosis and stenosis, has rarely been examined in a systematic manner. With the expanding use of CTV in clinical practice, the opportunity to investigate this relationship objectively is increasing. Such analysis is essential to deepen our understanding of the vascular consequences of overweight and obesity and to provide a scientific basis for targeted body weight management and preventive care. This study contributed foundational evidence to a field where population-based data remain scarce. CTV, with its superior capacity to visualize LEDV structures, provides a valuable means of assessing how excess adiposity contributes to venous remodeling and thrombotic processes.

Although BMI does not capture body composition in detail, it remains quite practical and standardized measure for categorizing weight status in retrospective analyses. Based on the available medical record conditions, BMI was adopted in this study, applying the widely recognized Chinese population standards. Previous reports on LEDV status assessed through CTV were mostly isolated case descriptions. Therefore, the present analysis of 349 cases accumulated over several years represented a substantial and rare dataset, providing a reliable foundation for exploring body weight-venous health associations. The inclusion of both male and female patients allowed for meaningful sex comparisons. The rarity of CTV-based dataset analyses in this area gave this study both originality and foundational importance.

Taken together, the interpretation of this study should be anchored in the multivariable analyses, rather than unadjusted crude comparisons. After accounting for established confounders including age, sex, hypertension, diabetes, hyperlipidemia, and documented major venous thromboembolism risk factors, BMI showed no significant independent association with the prevalence of thrombosis, stenosis, or their combination (all *P* > 0.05), nor with lesion counts. Accordingly, the high unadjusted prevalence of LEDV abnormalities observed among overweight and obese patients may be partially attributed to age and coexisting comorbidities rather than an independent effect of adiposity. In contrast, male sex was independently associated with a greater number of stenoses and with overall lesion burden, whereas hypertension only exhibited a borderline association with stenosis count (Table 6). This pattern suggests that, rather than being more likely to develop stenosis, men tend to accumulate more stenotic lesions once disease is present, implying that sex influences the extent of disease more than its occurrence.

Before statistical adjustment, the descriptive associations were as follows. The overweight and obesity rate in patients with LEDV thrombosis and/or stenosis were 86.4 and 79.0% for males and females respectively (Table 3), which were considerably higher than the general adult obesity rate (above 50%) in China (26). In reverse, 65.1 and 61.5% male and female overweight or obese patients suffered from LEDV abnormalities (Tables 1, 3). This bidirectional correlation revealed a strong positive association between excess body weight and venous pathology, reinforcing a clinically meaningful link. These findings suggested that obesity was associated with LEDV disease in this cross-sectional sample. A bidirectional relationship, in which LEDV pathology might in turn limit physical activity and contribute to further weight gain, is biologically plausible but cannot be established by the present design; its direction and temporality therefore remain hypotheses. This association nonetheless supports attention to weight management in this population.

However, unexpectedly, the prevalence of LEDV thrombosis (expressed as the average number of lesions per person) among overweight and obese male patients was slightly lower than that among those with normal or low body weight, though this difference was not statistically significant (*P* > 0.05). A similar but smaller pattern was observed in females. Among females aged 40 years and older, and particularly those over 60 year-old, the combined prevalence of thrombosis and/or stenosis was somewhat higher in overweight and obese groups without statistical significance (*P* > 0.05). For LEDV stenosis, however, a significant increase was found in overweight and obese males compared with normal-weight males (*P* < 0.05), most notably in the ≥60-year group. Female patients demonstrated a comparable but less pronounced trend. Overall, the combined prevalence of thrombosis and/or stenosis was higher in overweight and obese individuals especially the people over 60 than in normal-weight subjects, though the difference did not reach statistical significance (*P* > 0.05). These observations indicated that the association between BMI and LEDV disease might not be linear. Instead, the effect appeared to be shaped by multiple modifiers such as sex, age, comorbid conditions, and the chronicity of obesity. The higher rate of stenosis among people over 60 obese males could reflect long-term venous hypertension and structural remodeling resulting from sustained hemodynamic stress.

The relationship between BMI and LEDV abnormalities in this study appeared to be influenced by the coexistence of metabolic comorbidities obviously including hypertension, diabetes, and hyperlipidemia, as well as by sex. When these conditions were present, the prevalence of LEDV thrombosis in Group A was significantly higher in females than in normal-weight patients with similar comorbidities (*P* < 0.05). The prevalence of LEDV stenosis was also elevated in both male and female Group A patients compared to their normal-weight counterparts, with a larger difference observed among males, though not statistically significant (*P* > 0.05). When thrombosis and stenosis were considered together, Group A patients of both sexes demonstrated higher prevalence than Group B patients, indicating a consistent upward trend. These findings aligned with large cohort data showing that the presence of even a single component of metabolic syndrome independently increased the risk of VTE recurrence, and that the risk rose cumulatively with each additional component (27).

Especially, in females, obesity accompanied by hypertension, diabetes, and/or hyperlipidemia appeared to create a particularly prothrombotic environment likely due to systemic inflammation, insulin resistance, and hormonal influences that foster hypercoagulability. This interpretation was supported by large-scale studies demonstrating that obesity-related parameters exerted a greater effect on the risk of deep vein thrombosis and pulmonary embolism in women than in men (28). Collectively, these data highlighted a synergistic interaction between metabolic dysfunction and female in amplifying venous pathology.

Sex differences emerged as a prominent theme in this analysis. Among men, overweight and obesity even with metabolic comorbidities, did not correspond to a higher prevalence of LEDV thrombosis compared with normal-weight groups, in contrast to the pattern observed in women. Interestingly, in males, the prevalence of LEDV thrombosis was actually higher in normal-weight than in overweight or obese individuals, though this difference lacked statistical significance. In both sexes, overweight and obese patients exhibited more LEDV stenosis than normal-weight ones, but the increase was more substantial in men, some differences reaching significance. After stratifying by comorbidity status, an inverse trend appeared in men: Group A males had less LEDV thrombosis than Group B males, whereas females showed the opposite. Stenosis remained higher in Group A males than in females (*P* < 0.05). This paradoxical pattern may reflect the confounding effect of medication use, whereby prescribed anticoagulant or antiplatelet agents for cardiovascular prevention could inadvertently reduce the risk of venous thrombosis, thereby masking true associations.

Indeed, recent evidence suggested that obese patients anticoagulated for non-valvular atrial fibrillation with xabans or warfarin achieved greater protection from thrombosis than their non-obese counterparts (29). Together, these suggested that men and women might follow distinct pathophysiological pathways to LEDV disease, one influenced more by pharmacologic and hemodynamic factors, the other by hormonal and metabolic mechanisms.

Our study also illustrated sex-specific value of preventive strategies. Women, particularly those who are overweight or obese and have metabolic comorbidities, appeared to derive greater potential benefit from proactive weight management in reducing LEDV thrombosis risk. The positive association between BMI and LEDV abnormalities in women strengthened the rationale for incorporating body weight control into venous health programs. Given that 36.7% of the patients in this study had hypertension, diabetes, and/or hyperlipidemia, extrapolating this pattern to the broader population suggests a significant public health impact. Early identification and management of overweight/obesity, particularly earlier in life, may be relevant to LEDV burden; however, as this analysis is cross-sectional, whether such intervention reduces the incidence or recurrence of LEDV thrombosis and stenosis remains a hypothesis to be tested prospectively.

This study, while exploratory, provided valuable preliminary evidence but was not without limitations. Importantly, this is a retrospective cross-sectional analysis of patients who had already undergone CTV; it therefore cannot establish a temporal sequence or causation between elevated BMI and LEDV abnormalities, and all relationships reported above should be interpreted as associations rather than risk or causal effects. In addition, because all participants were hospitalized patients clinically referred for CTV, they already carried a high pre-test probability of venous pathology; this referral (Berkson) selection bias means the prevalence estimates and associations observed here may not generalize to the general population, and our conclusions are accordingly restricted to comparable hospitalized populations undergoing clinically indicated CTV. Moreover, even after adjusting for major venous thromboembolism risk factors that could only be entered as a sparse, documented binary composite (yielding wide confidence intervals), several other clinically important confounders, including hormonal, anticoagulant, or antiplatelet therapy and the acute event-to-imaging interval, remained unrecorded. Residual confounding from these unmeasured factors cannot be fully excluded and may disproportionately affect the sex-related findings. Although 349 cases from a Class A tertiary hospital were analyzed over seven consecutive years, a robust sample for a CTV-based study, the sample size might still have been insufficient to achieve statistical significance due to the large variance in the number of LEDV thrombosis and stenosis lesions per capita. Besides, Yuan S et al. have reported that waist circumference might better reflect the link between obesity and VTE risk than BMI (30). Thus, the multivariable findings should be regarded as hypothesis-generating and warrant confirmation in larger, prospective cohorts to inform evidence-based strategies for weight and venous health management.

## 5 Conclusion

Higher BMI was associated with a greater prevalence of CTV-detected LEDV abnormalities, particularly stenosis and in the presence of comorbidities. After multivariable adjustment, however, BMI was not an independent predictor; male sex was the principal independent correlate of stenosis burden, with hypertension borderline. These cross-sectional associations are hypothesis-generating.

## Data Availability

The original contributions presented in the study are included in the article/supplementary material, further inquiries can be directed to the corresponding author/s.
